# Phenological synchrony between eastern spruce budworm and its host trees increases with warmer temperatures in the boreal forest

**DOI:** 10.1002/ece3.4779

**Published:** 2018-12-21

**Authors:** Deepa S. Pureswaran, Mathieu Neau, Maryse Marchand, Louis De Grandpré, Dan Kneeshaw

**Affiliations:** ^1^ Canadian Forest Service Laurentian Forestry Centre Québec City Québec Canada; ^2^ Département des Sciences Biologiques Université du Québec à Montréal Montréal Québec Canada

**Keywords:** climate change, disturbance, insect outbreaks, plant‐insect interactions, range shifts

## Abstract

Climate change is predicted to alter relationships between trophic levels by changing the phenology of interacting species. We tested whether synchrony between two critical phenological events, budburst of host species and larval emergence from diapause of eastern spruce budworm, increased at warmer temperatures in the boreal forest in northeastern Canada. Budburst was up to 4.6 ± 0.7 days earlier in balsam fir and up to 2.8 ± 0.8 days earlier in black spruce per degree increase in temperature, in naturally occurring microclimates. Larval emergence from diapause did not exhibit a similar response. Instead, larvae emerged once average ambient temperatures reached 10°C, regardless of differences in microclimate. Phenological synchrony increased with warmer microclimates, tightening the relationship between spruce budworm and its host species. Synchrony increased by up to 4.5 ± 0.7 days for balsam fir and up to 2.8 ± 0.8 days for black spruce per degree increase in temperature. Under a warmer climate, defoliation could potentially begin earlier in the season, in which case, damage on the primary host, balsam fir may increase. Black spruce, which escapes severe herbivory because of a 2‐week delay in budburst, would become more suitable as a resource for the spruce budworm. The northern boreal forest could become more vulnerable to outbreaks in the future.

## 1 INTRODUCTION

In temperate climates, the degree of synchrony between herbivorous insects and their host plants is crucial to the completion of insect life cycles (Van Asch & Visser, 2007). Recent studies on forest responses to warming predict extension of the growing season with earlier springs and delayed winters (Gunderson et al., 2012). Northern range limits of temperate and boreal species are determined by the duration of the growing season and winter temperature (Bale et al., 2002). Therefore, plants and defoliating insects are highly sensitive to changes in temperature that trigger, respectively, budburst and emergence from diapause (Beck, 1983; Logan, Régnière, & Powell, 2003). Climate change has also been implicated in altering the seasonal phenology of trees, herbivores, and natural enemies at different rates (Singer & Parmesan, 2010; Stireman et al., 2005; Voigt et al., 2003), thereby changing species distributions (Chuine, 2010; Hill, Griffiths, & Thomas, 2011) as well as interspecific interactions. As a result, changes have recently been observed in the distribution of plants and their insect herbivores including shifts to new hosts and consequently, in the disturbance regimes of pest species (Haynes, Allstadt, & Klimetzek, 2014; Jepsen, Hagen, Ims, & Yoccoz, 2008; Jepsen et al., 2011).

The recognition of phenology as an important adaptive trait shaping species distributions requires examination of responses to temperature by both plants and their insect herbivores. Among North American and European pest species that cause outbreaks, landscape‐level consequences of phenological changes are becoming more pronounced. In European Lepidoptera, examination of variation in phenological change of 566 species over a 150‐year period suggests that shifts in phenology related to climate change are correlated with traits involving interactions with host plants (Altermatt, 2010 and references therein). Among forest defoliators, recent changes in distribution patterns of the winter moth, *Operophtera brumata* (Hagen, Jepsen, Ims, & Yoccoz, 2007) and the autumnal moth, *Epirrita autumnata* (Jepsen et al., 2008) have been reported. In North America, flexible voltinism (from semivoltine to univoltine) in response to temperature has been recorded in the spruce beetle, *Dendroctonus rufipennis* (Hansen & Bentz, 2003; Hansen, Bentz, Powell, Gray, & Vandygriff, 2011), particularly in the northern part of its range, increasing severity of outbreaks in recent years (Berg, Henry, Fastie, Volder, & Matsuoka, 2006; Werner & Holsten^1985^, ^1985^; Werner, Holsten, Matsuoka, & Burnside, 2006). As predicted by Williams and Liebhold (2002), there are now instances of outbreaks of the southern pine beetle, *Dendroctonus frontalis*, in more northern pine forests in the United States that they attribute to an increase in minimum winter temperatures (Tran, Ylioja, Billings, Régnière, & Ayres, 2007; Weed, Ayres, & Hicke, 2013).

Similarly, the current outbreak of an important defoliator in the eastern North American boreal forest, the eastern spruce budworm, *Choristoneura fumiferana,* is occurring farther north than in the past, and in areas that were not previously affected by outbreaks (Pureswaran et al., 2015). Spruce budworm is a univoltine moth whose larvae feed on current year buds of balsam fir (*Abies balsamea* (L.) Mill.) and black spruce (*Picea mariana* (Mill.). Larvae emerge from diapause in the spring and disperse on silk to settle on new buds. Budburst of balsam fir, the preferred host, may occur up to two weeks following larval emergence, in which case, larvae may mine old foliage while waiting for budburst. A lag that is too long between larval emergence and budburst results in high rates of larval mortality (Blais, 1957; Nealis & Régnière, 2004; Trier & Mattson, 1997). Synchrony with budburst and its consequences on survival were recorded to be optimal when larval emergence preceded budburst by about 2 weeks on white spruce (Lawrence, Mattson, & Haack, 1997), whose budburst occurs at the same time as balsam fir (Greenbank, 1963). Emerging too late in relation to budburst also reduces fitness as lignified foliage is difficult for young larvae to feed on (Lawrence et al., 1997). Recent studies that manipulated the availability of new buds to second instar larvae showed that phenological asynchrony with both balsam fir and black spruce has a negative impact on spruce budworm performance (Fuentealba, Pureswaran, Bauce, & Despland, 2017; Fuentealba, Sagne, Pureswaran, Bauce, & Despland, 2018).

The northern boreal forest is composed of balsam fir as well as a high proportion of black spruce. White spruce is a minor component in this region and is grouped with balsam fir in forest inventories. Black spruce is usually a secondary host species, that in the past suffered little mortality from defoliation for two reasons: (a) the cool, short summers that characterize this northern ecosystem limited completion of spruce budworm's life cycle; (b) budburst of black spruce occurs two weeks later than that of balsam fir (Nealis & Régnière, 2004). The 3–4 week lag between budburst of black spruce and larval emergence from diapause has historically been sufficient to allow black spruce to phenologically escape from severe herbivory during outbreaks. However, during the current outbreak, we documented significant defoliation of black spruce (Bognounou, Grandpre, Pureswaran, & Kneeshaw, 2017), raising concerns about an increase in the severity of outbreaks if an advance in budburst due to climate warming were to render black spruce phenologically better suited to spruce budworm.

Several studies in Europe and North America have examined the role of climate change in relation to tree phenology (Menzel et al., 2006; Wolkovich et al, 2012). Other studies have looked at the role of temperature in relation to insect developmental rates (Régnière, Powell, Bentz, & Nealis, 2012). Fewer studies have examined the impact of temperature on both plant and insect phenology, including changes in phenological synchrony and mismatches due to simulated climate warming (Buse & Good, 1996; Schwartzberg et al., 2014). Still fewer studies have been conducted in nature to evaluate in situ variation in phenology between forest insects and their host trees and their response in the long‐term to warmer ambient temperatures. In the midst of a bourgeoning spruce budworm outbreak (Ministère des Forêts & de la Faune et des Parcs, 2016), we used naturally occurring microclimates in mixed stands of black spruce and balsam fir to test the following predictions: (a) warm microclimates will advance budburst of balsam fir and black spruce, (b) warm microclimates will also advance the emergence of spruce budworm larvae from diapause, and (c) if there are advances in budburst and larval emergence, they will occur at different rates, potentially increasing phenological synchrony between spruce budworm and both or one of its host species.

## 2 METHODS

### 2.1 Study sites and sampling design

The study area is located in the transition between meridional balsam fir‐dominated boreal forests to more northern black spruce‐dominated forests in eastern Quebec, Canada (Saucier, Robitaille, Grondin, Bergeron, & Gosselin, 2011). A cold maritime climate characterizes this region, where mean annual temperature varies from −2.0 to 1.5°C and mean annual precipitation ranges from 950 to 1,350 mm, of which about 40% falls as snow. The growing period lasts 120 to 150 days (Saucier et al., 2009).

We established two sites in the midst of an ongoing spruce budworm outbreak, 50 km north of the town of Baie‐Comeau, one in 2013 (49°43′46.84″N, −68°10′8.76″W) and the other in 2014 (49°42′59.00″N, −68°8′26.30″W) in mixed black spruce/balsam fir stands older than 90 years. Balsam fir and black spruce about three meters in height were selected at each site with 20 trees of each species in 2013 and 30 in 2014. Trees were chosen to represent a gradient of microclimates. Average defoliation in the region of our study was 50% in 2012, 64% in 2013, and 75.8% in 2014. The average defoliation of balsam fir and black spruce trees reached 86.3% and 46.7%, respectively, in the summer of 2013 inhibiting bud production by balsam fir trees the following year. We had to change study plots in 2014 because severe defoliation by the spruce budworm made it impossible to collect data from the same trees for more than one summer.

### 2.2 Phenology measurements

**Figure 1:**
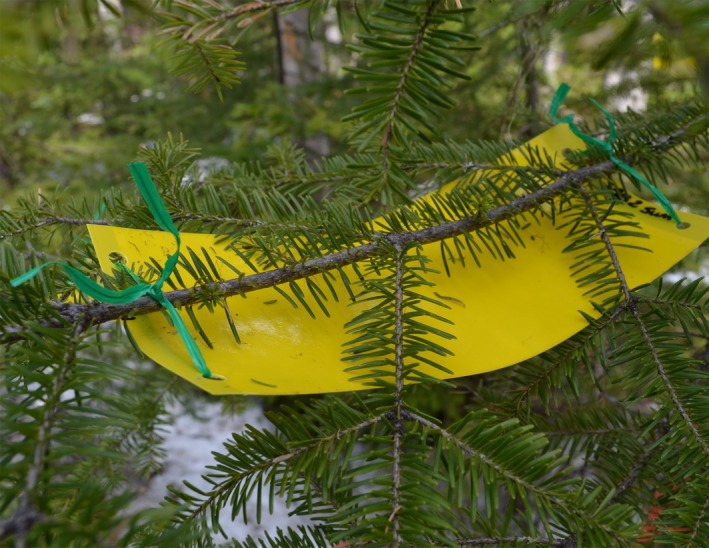
Capture of spring emergence of overwintering second instar larvae (L2) of *Choristoneura fumiferana *using sticky traps. Traps were installed 2–3 cm below selected branches to ensure that a majority of larvae captured had overwintered in the branches directly above

We monitored the onset of budburst and emergence of second instar larvae (L2) from diapause on all trees. Every 2–3 days, 30 or 50 buds, in 2014 and 2013 respectively, were randomly selected on branches located around each tree to observe budburst. Buds were classified as “open,” when bud scales started to separate and the tips of needles were visible (Dorais & Kettela, 1982; Numainville & Desponts, 2004), or “closed” otherwise. Yellow “dry‐touch” sticky traps (Solida, Saint‐Ferréol‐les‐Neiges, QC, Canada) were installed a 2–3 cm below selected branches (Figure 1) to capture larvae as they emerged from diapause and before they dispersed in search of food. Because of the proximity of the sticky traps to the selected branches, it was highly likely that the larvae we captured emerged from the branch directly above the trap (Figure 1). Traps were monitored every 2–3 days until no more larvae were collected. The onset of budburst was defined as the date when at least one bud was open on the tree, and larval emergence was estimated at peak emergence, when the maximum number of larvae was observed on each tree. Synchrony was calculated as the time difference (lag, in days) between these two phenological events.

### 2.3 Climate data

Temperature data were recorded hourly between May and July each year. HOBO Pendant^®^ (UA‐002–64, Onset Computer Corporation, Bourne, MA, USA) or Thermochron iButton^®^ (DS1921G, Maxim Integrated Products, Inc., San Jose, CA, USA) temperature data loggers were installed on each tree from which phenology data were collected. Sensors were placed on a south facing branch at a height of about 1.30 m. Based on data availability, a common reference period was designated (Julian days 155–173) and hourly measurements taken during that period were averaged for each tree. This average temperature was used to define microclimates and compare trees within the same site (year).

In order to estimate spring temperatures before the beginning of our sampling, daily air temperature was interpolated for our sites with BioSIM 10 software (Régnière, 1996; Régnière & St‐Amant, 2007; Régnière, St‐Amant, & Bechard, 2014), using the daily climatic model (Régnière & Bolstad, 1994). BioSIM interpolates daily minimum and maximum temperatures (°C), precipitation (mm), relative humidity, and wind speed by matching georeferenced sources of weather data (eight nearest weather stations) to spatially georeferenced points (study sites), adjusting for differences in latitude, longitude, and elevation between the source of weather data and each site location using spatial regressions (Régnière et al., 2014).

### 2.4 Statistical analysis

**Figure 2:**
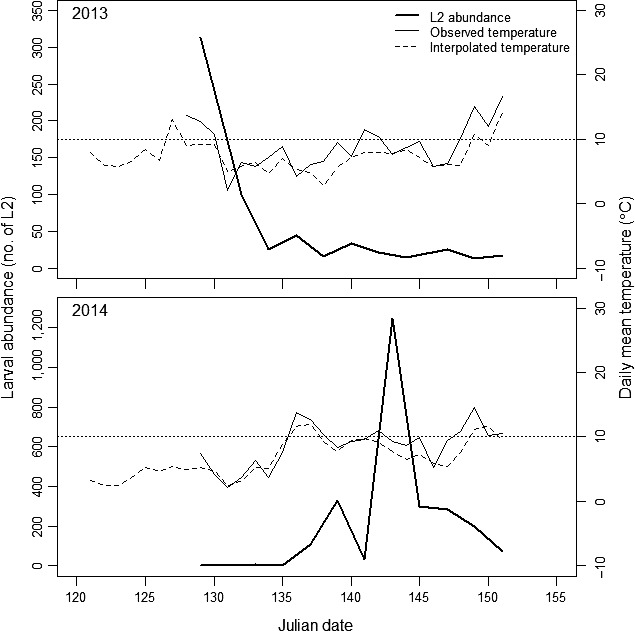
Spring emergence of overwintering second instar larvae (L2) of *Choristoneura fumiferana* over time, in May 2013 (top) and 2014 (bottom). Bold solid lines represent total number of L2. Dashed lines show interpolated daily mean air temperatures and thin solid lines show observed daily mean temperatures (microclimates), averaged over all trees. Sticky traps were installed on Julian days 127 and 129, in 2013 and 2014 respectively. Horizontal dotted lines indicate the 10°C temperature threshold, above which larvae started to emerge (see section on “Larval emergence from diapause” in Results for explanation)

**Figure 3:**
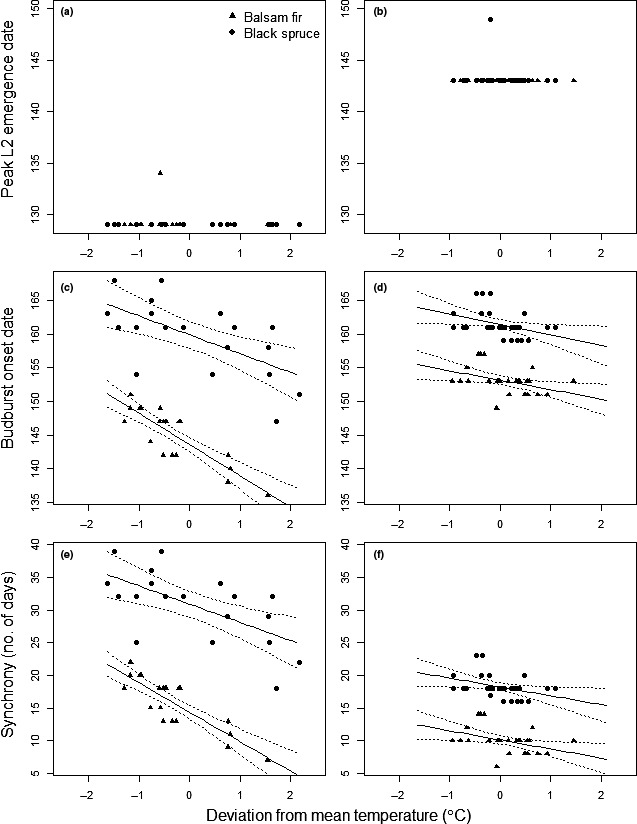
Relationships between onset of second instar larval (L2) emergence (a, b), onset of budburst (c, d), and synchrony (e, f) with mean‐centered microclimate temperatures, for 2013 (left column; *n* = 39) and 2014 (right column; *n* = 59). Solid lines show model predictions in the observed range of temperatures. Dotted lines are 95% confidence intervals. No model was fitted to L2 emergence data but observed values are plotted to show the relationship between the two phenological events and synchrony

The influence of temperature on budburst onset and phenological synchrony was assessed using linear regression models. Regression analysis was also initially used to evaluate the relationship between peak larval emergence and temperature. However, peak larval emergence was synchronous, that is, on the same day in a given year on most trees, regardless of temperature (Figures 2 and 3a,b; i.e., day 129 in 2013 and day 143 in 2014). Therefore, we did not perform regression analysis on larval emergence data.

A single model containing microclimates, species, site, and their interactions was adjusted to both dependent variables, that is, budburst onset and phenological synchrony. Models were parametrized to provide slope estimates for each group (species x site) and their appropriate standard errors (Schielzeth, 2010). Temperature was mean‐centered before analyses to facilitate interpretation of intercept terms, and residuals were plotted to verify model assumptions. The *glht* function of the *multcomp* package (Hothorn, Bretz, & Westfall, 2008) was then used to simultaneously perform slope comparison tests between species and sites (years). Two trees were removed before analyses, one black spruce tree in 2013 was too severely defoliated to follow budburst, and another black spruce in 2014 had too few larvae on it to estimate peak emergence.

Preliminary ordinary least squares regressions showed unequal variance in the residuals between groups, violating the assumption of homoscedasticity. Generalized least squares (GLS) regressions were performed, using the *gls* function of the *nlme* package (Pinheiro, Bates, DebRoy, Sarkar, & Team, 2016) in R (R Core Team, 2016). This procedure allows for heteroscedastic within‐group errors by directly modeling the variance–covariance structure of the response (Pinheiro & Bates, 2000). Different variance structures were tested and compared using AICc (Akaike's information criterion) corrected for small sample sizes (Burnham & Anderson, 2002) using the *AICc* function in the *AICcmodavg *package (Mazerolle, 2016). Models with the *varIdent* variance function (Pinheiro & Bates, 2000), allowing for different variances for each group, provided the best fit. Examination of standardized residuals further showed that heteroscedasticity was properly accounted for.

## 3 RESULTS

### 3.1 Microclimates and air temperature

**Figure 4:**
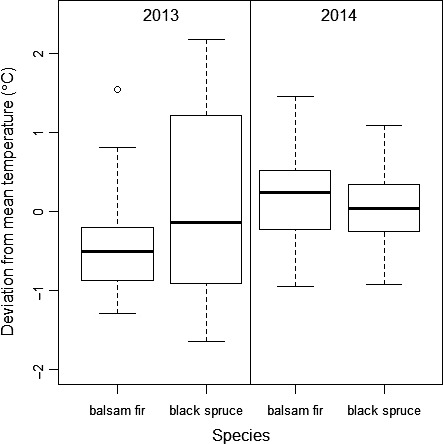
Distribution of mean‐centered microclimate temperatures recorded in the 2013 site and 2014 site during the reference period (Julian days 155–173) on sampled trees of two species, *Abies balsamea* and *Picea mariana*. Data from all days were averaged for each tree

We found a maximum temperature difference of 3.82°C between the coldest and warmest microclimates, sampled across sites (years) and species (Figure 4). The range was greater in 2013 (2.83°C for balsam fir and 3.82°C for black spruce) than in 2014 (2.4°C for balsam fir and 2.01°C for black spruce). Climate records from the province of Quebec indicate an increase of 1–3°C of mean annual temperatures over a period of between 1950–2011. This trend is expected to continue so that annual temperatures rise by approximately 2–4°C for the 2041–2070 period (Ouranos, 2015). The temperature range in our study therefore reflects predicted warming scenarios. Average air temperatures interpolated for each site were consistent with average observed microclimate temperatures and show that the period preceding the phenological events was colder in 2014 than in 2013 (Figure 2).

### 3.2 Larval emergence from diapause

A total of 3,394 second instar larvae (L2) were sampled during the study; 300 on black spruce and 377 on balsam fir in 2013, and 893 on black spruce and 1,824 on balsam fir in 2014. Peak emergence of L2s was neither influenced by microclimate nor host species (Table 1, Figure 3a,b). Larval emergence peaked on the same date for all but one tree in both years (Figure 3a,b). In 2013, peak emergence occurred on day 129 except for one balsam fir (day 134); in 2014, larvae emerged on day 143 (two weeks later) except for one black spruce (day 149). A few larvae had emerged before we started collecting our samples in 2013, but it is highly likely that our sampling period captured peak larval emergence. According to interpolated air temperatures before larval sampling began, a mean temperature of 10°C was not attained before day 127 when our sticky traps were installed (Figure 2). Based on the 2014 results, larval emergence appears to occur once average air temperatures reach 10°C. On day 136, when temperatures reached 10°C, a few larvae began to emerge (Figure 2). Three days later, temperatures dropped below this threshold and the number of sampled larvae dropped correspondingly. When temperatures reached 10°C again around day 142, peak emergence occurred.

**Table 1:** Characteristics of sampled trees. Values are means with standard deviation in parentheses

	2013		2014		Overall
	Balsam fir	Black spruce	Balsam fir	Black spruce	
Sample size	20	19	30	29	98
L2 emergence onset date	129.3 (1.1)	129 (0)	143 (0)	143.2 (1.1)	137.5 (6.9)
Budburst onset date	145.1 (4.1)	159.6 (5.5)	152.9 (1.9)	161.5 (1.9)	155.1 (7.0)
Lag (no. of days)	15.9 (4.0)	30.6 (5.5)	9.9 (1.9)	18.2 (1.8)	17.6 (7.9)

### 3.3 Budburst phenology

**Figure 5:**
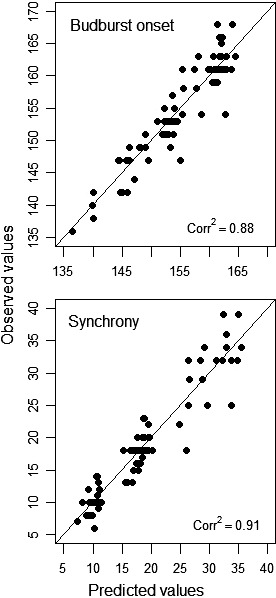
Relationship between predicted and observed values, for the budburst onset and synchrony generalized least squared (GLS) regression models. The straight line represents perfect agreement, and Corr^2^ indicates the squared correlation between the two

In 2013, the onset of budburst started on day 136 (May 16th) and ended on day 168 (June 17th), the onset period lasting 15 days for balsam fir and 21 days for black spruce. In 2014, the budburst period started almost two weeks later (day 149—May 29th) but was shorter (17 days overall, 8 days for balsam fir and 7 days for black spruce) and ended earlier, on day 166 (June 15th). The timing of budburst advanced with increasing temperature for both species and in both years (Figure 3c,d, Table 2). The effect was strongest for balsam fir in 2013: budburst occurred on average 4.62 days earlier for an increase in temperature of 1°C. This represents a difference of almost two weeks (13 days) between the warmest and coldest microclimates observed for that group (with a temperature range of 2.83°C). In the same year, black spruce budburst onset advanced by 11 days (slope: −2.79, temperature range of 3.82°C). The influence of microclimate temperature was lower in 2014, with budburst advancing by only three days for between the warmest and coolest microclimates for both species (slopes: −1.39 and −1.53, ranges: 2.40 and 2.01°C, for balsam fir and black spruce, respectively). The GLS model provided a good fit to the data (Figure 5). Slope comparisons among groups were not statistically significant, except for balsam fir when compared between years (Table 3). This is due to large confidence intervals around estimates. In 2013, the onset of budburst occurred 16 days earlier, at mean microsite temperature, for balsam fir compared to black spruce (Table 2). The difference between species was half this value in 2014 (8 days), with balsam fir budburst occurring almost 10 days later than in 2013.

**Table 2:** Parameter estimates from generalized least squared (GLS) regression models

	GLS models	
	Budburst onset	Synchrony
Mean for balsam fir 2013	143.6*** (142.5, 144.7)	14.4*** (13.3, 15.4)
Difference in means (days)		
Balsam fir 2014	9.6*** (8.3, 10.8)	−4.2*** (−5.5, −3.0)
Black spruce 2013	16.3*** (14.0, 18.6)	16.5*** (14.3, 18.8)
Black spruce 2014	17.9*** (16.6, 19.1)	3.9*** (2.7, 5.1)
Effect of temperature		
Balsam fir 2013	−4.6*** (−5.9, −3.3)	−4.5*** (−5.8, −3.2)
Balsam fir 2014	−1.4* (−2.6, −0.2)	−1.4* (−2.6, −0.2)
Black spruce 2013	−2.8*** (−4.4, −1.1)	−2.8*** (−4.4, −1.1)
Black spruce 2014	−1.5* (−2.9, −0.2)	−1.4* (−2.6, −0.1)
Residual standard error	4.4	4.4
Corr^2^	0.88	0.91
Number of observations	98	98

Note

Values in parentheses are 95% confidence intervals. Corr^2^ indicates the squared correlation between predicted and observed values. Since temperature was mean‐centered before analyses, intercept terms represent mean at average microsite temperature and comparisons with the reference group (balsam fir 2013). Model was parametrized to provide group slope estimates, that is, a temperature slope for each species and site (year). Onset of budburst and synchrony are measured in number of days.

Asterisks indicate statistical significance:

*p < 0.05; **p < 0.01; ***p < 0.001

**Table 3:** Simultaneous tests for general linear hypotheses (slope comparisons). Values in parentheses are 95% confidence intervals. Onset of budburst and synchrony are measured in number of days

Slope comparisons	Budburst onset	Synchrony
Balsam fir 2013 versus Balsam fir 2014	3.2** (1.0, 5.4)	3.1** (0.9, 5.3)
Black spruce 2013 versus Black spruce 2014	1.3 (−1.4, 3.9)	1.4 (−1.1, 4.0)
Balsam fir 2013 versus Black spruce 2013	1.8 (−0.8, 4.5)	1.7 (−0.9, 4.3)
Balsam fir 2014 versus Black spruce 2014	−0.1 (−2.4, 2.1)	0.04 (−2.1, 2.2)

Note

Asterisks indicate statistical significance:

*p < 0.05; **p < 0.01; ***p < 0.001

### 3.4 Synchrony between budburst onset and peak larval emergence

In 2013, the number of days between the onset of budburst and peak larval emergence ranged from 7 to 22 days for balsam fir and 18 to 39 days for black spruce. The phenological events were more synchronous in 2014 when the number of days between budburst and larval emergence varied from 6 to 14 days on balsam fir and 16 to 23 days for black spruce.

Peak larval emergence is practically the same for all trees sampled in the same year and thus differences in synchrony are due primarily to the onset of budburst (Figures 2 and 3; Tables 2 and 3). Comparing microclimates, synchrony was 13 and 11 days closer in 2013, for balsam fir and black spruce, respectively in the warmest versus the coolest microclimates. The difference between species was not statistically significant (Table 3). In 2014, synchrony increased by only three days for both species between the minimum and maximum observed temperatures, but the length of the budburst period was also shorter.

## 4 DISCUSSION

Our study has shown that under natural conditions, while larval emergence responds to a rise in average daily air temperature above 10°C (Figure 2), budburst in both host species advances significantly for every degree rise in microsite temperature (Figure 3c,d, Table 2). An advance in budburst, and as a consequence, tighter phenological synchrony between spruce budworm and its hosts (Figure 3, Table 2), can potentially decrease larval mortality due to predation or starvation that occurs during the waiting period, particularly on balsam fir, the primary host of spruce budworm. Black spruce, that is currently less accessible as a host (Nealis & Régnière, 2004), would become more suitable to emerging second instar larvae. Lawrence et al. (1997) noted that spruce budworm larval survival on white spruce, *Picea glauca*, was greatest when larval development began about two weeks before budburst, presumably because early budburst and foliage maturation may impede feeding, whereas, late budburst would lead to starvation of young larvae. We expect that the “phenological window” of host suitability could potentially increase for both balsam fir and black spruce as phenological synchrony becomes tighter.

The effects of temperature on advance in budburst (Figure 3c,d) and correspondingly on synchrony (Figure 3e,f) were more pronounced in 2013 than in 2014 because of the wider range of temperatures that prevailed in the microclimates of our sampled trees in 2013 (Figure 4). In spite of this difference in variation in temperature between years, budburst and synchrony advanced with temperature in both years (Figure 3, Table 2). Similar changes in budburst phenology were recently observed by Rossi (2015) who showed in greenhouse experiments that one‐year‐old black spruce seedlings from cold sites that were subjected to warm temperatures had earlier budburst. In the southern part of its range, black spruce budburst occurs when temperatures are between 9 and 13°C (Huang, Deslauriers, & Rossi, 2014). Within this range of temperatures, a slight warming could significantly advance budburst and create changes in the phenological synchrony between interacting species. The phenological delay between black spruce and spruce budworm, and as a consequence, the diminished performance and low population densities on this host species, were first reported by Swaine and Craighead (1924). Blais (1957) further documented that black spruce as a food resource had no adverse effect on the rate of larval development nor survival of spruce budworm larvae and concluded that phenological delay in budburst was the main factor that rendered black spruce relatively resistant to defoliation. More recent work has shown that while survival of early stages of spruce budworm may be low on black spruce due to its delayed budburst, survival of late instar larvae was relatively high as phenological differences among hosts decreased as the summer season progressed and foliage on all hosts became equally suitable (Nealis & Régnière, 2004).

Our prediction that emergence of second instar larvae from diapause would also advance under warmer temperatures did not hold true over the temperature gradient we observed. Larval emergence did not exhibit any discernable relationship with neither temperature nor host species (Figure 3a,b, Table 1). Instead, maximum larval emergence occurred once air temperature exceeded 10°C (Figure 2). Diapause in spruce budworm is completed by the end of February after which larvae remain quiescent, waiting for suitable environmental cues such as temperature and photoperiod before they emerge to continue development (Bean, 1961). Development rates during the post‐diapause period were found to increase at temperatures above 11°C (Régnière, 1990). Similarly, in a study of the response of the western spruce budworm, *Choristoneura occidentalis*, to temperature, Reichenbach and Stairs (1984) noted that development of all life stages was minimal below 10°C. Régnière (1987) also found that low temperatures delayed larval emergence.

The prolonged post‐diapause development period of overwintering larvae (Bean, 1961) usually makes it difficult to predict timing of larval emergence in the spring based on developmental physiology (Régnière, 1990). Emergence under field conditions can peak, with larvae emerging more simultaneously than was accounted for by variability in developmental rates and often occurred earlier than models predicted (Lysyk, 1989). Fluctuating temperatures can also synchronize post‐diapause development in the population (Régnière, 1987) and lead to simultaneous emergence of larvae as we observed in our study. There are several benefits to simultaneous emergence in overwintering larvae, particularly in northern latitudes with relatively short summers, because they need to take advantage of environmental conditions as soon as they become favorable.

Emerging from diapause or hatching too far in advance of budburst incurs several costs, and global warming is likely to disrupt existing phenological relationships. When subjected to warm spring temperatures, egg hatch of the winter moth was predicted to occur up to three weeks before budburst of pedunculate oak, *Quercus robur, *becoming poorly synchronized as a result (Visser & Holleman, 2001). In general, tighter phenological synchrony with host plants has a positive impact on the population dynamics of defoliating Lepidoptera and can potentially increase outbreak severity (Van Asch & Visser, 2007). Trends similar to those observed in our study in which host tree phenology advanced more than insect phenology in response to temperature, thereby decreasing the lag between egg hatch and budburst of hosts and improving synchrony, have also been observed for the forest tent caterpillar, *Malacosoma disstria* (Schwartzberg et al, 2014).

Demonstrating the precise impacts of climate change on natural ecosystems, particularly across trophic levels is a challenging task (Mjaaseth, Hagen, Yoccoz, & Ims, 2005; Parmesan & Yohe, 2003). Most current information on relative changes between host budburst and insect emergence is from experimental manipulation or predictive models (Harrington, Woiwood, & Sparks, 1999). Our study is the first to measure emergence from diapause in the field for the spruce budworm in association with budburst phenology and temperature. From a practical standpoint, one of the challenges we encountered was that emergence from diapause often occurred earlier than models predicted (Lysyk, 1989) while there was still over a meter of snow on the forest floor. Phenological traits are also reported to be highly heritable (Chuine, 2010) and are subject to rapid selection in insects. However, there are not many empirical studies evaluating natural selection on phenological traits over several generations. So far, the winter moth–pedunculate oak system is the only forest insect for which the potential to adapt rapidly to changing host phenology, restoring synchrony of egg hatch with host budburst has been demonstrated (Van Asch, Tienderen, Holleman, & Visser, 2007). To predict the impacts of climate change in forest pest systems, it will be crucial to determine the relative speed of adaptation at different trophic levels and the consequences for population dynamics (Forrest & Miller‐Rushing, 2010; Harrington et al., 1999). In the spruce budworm system, an increase in synchrony between both host species and spruce budworm may increase the severity of outbreaks, particularly in mixed stands of black spruce and balsam fir (Bognounou et al., 2017). The “phenological window of host suitability” in this case, would widen by sustaining populations on black spruce later in the season, as well as during the course of the outbreak cycle after balsam fir is defoliated and larvae spill over to feed on black spruce. Long‐term studies over several generations are therefore required to determine whether the peak emergence dates of overwintering larvae might shift in adaptation to the budburst phenology of black spruce.

## AUTHOR CONTRIBUTIONS

DSP originally formulated the idea. DSP and LDG conceived and designed the study. MN performed the experiments and collected data. MM analyzed the data. DSP, MM, LDG, and DK wrote and edited the manuscript.

## DATA ACCESSIBILITY

Data and R‐scripts will be deposited upon acceptance of manuscript for publication in a publicly accessible repository such as Dryad or FigShare.
